# The IAP antagonist birinapant potentiates bortezomib anti-myeloma activity in vitro and in vivo

**DOI:** 10.1186/s13045-019-0713-x

**Published:** 2019-03-07

**Authors:** Liang Zhou, Yu Zhang, Yun Leng, Yun Dai, Maciej Kmieciak, Lora Kramer, Kanika Sharma, Yan Wang, William Craun, Steven Grant

**Affiliations:** 1 Division of Hematology/Oncology, Department of Medicine, Virginia Commonwealth University, P.O. Box 980035, Richmond, VA 23298 USA; 2 Department of Hematology, Beijing Chaoyang Hospital of Capital Medical University, Beijing, China; 3 Cancer Center, The First Hospital of Jilin University, Changchun, China; 4 Department of General Surgery, China-Japan Union Hospital of Jilin University, Changchun, China; 5 Massey Cancer Center, Virginia Commonwealth University Health Sciences Center, Richmond, VA USA

**Keywords:** Multiple myeloma, IAP antagonist, Bortezomib, NF-κB

## Abstract

**Background:**

Mechanisms by which Smac mimetics (SMs) interact with proteasome inhibitors (e.g., bortezomib) are largely unknown, particularly in multiple myeloma (MM), a disease in which bortezomib represents a mainstay of therapy.

**Methods:**

Interactions between the clinically relevant IAP (inhibitor of apoptosis protein) antagonist birinapant (TL32711) and the proteasome inhibitor bortezomib were investigated in multiple myeloma (MM) cell lines and primary cells, as well as in vivo models. Induction of apoptosis and changes in gene and protein expression were monitored using MM cell lines and confirmed in primary MM cell populations. Genetically modified cells (e.g., exhibiting shRNA knockdown or ectopic expression) were employed to evaluate the functional significance of birinapant/bortezomib-induced changes in protein levels. A MM xenograft model was used to evaluate the in vivo activity of the birinapant/bortezomib regimen.

**Results:**

Birinapant and bortezomib synergistically induced apoptosis in diverse cell lines, including bortezomib-resistant cells (PS-R). The regimen robustly downregulated cIAP1/2 but not the canonical NF-κB pathway, reflected by p65 phosphorylation and nuclear accumulation. In contrast, the bortezomib/birinapant regimen upregulated TRAF3, downregulated TRAF2, and diminished p52 processing and BCL-X_L_ expression, consistent with disruption of the non-canonical NF-κB pathway. TRAF3 knockdown, ectopic TRAF2, or BCL-X_L_ expression significantly diminished birinapant/bortezomib toxicity. The regimen sharply increased extrinsic apoptotic pathway activation, and cells expressing dominant-negative FADD or caspase-8 displayed markedly reduced birinapant/bortezomib sensitivity. Primary CD138^+^ (*n* = 43) and primitive MM populations (CD138^−^/19^+^/20^+^/27^+^; *n* = 31) but not normal CD34^+^ cells exhibited significantly enhanced toxicity with combined treatment (*P* < 0.0001). The regimen was also fully active in the presence of HS-5 stromal cells or growth factors (e.g., IL-6 and VEGF). Finally, the regimen was well tolerated and significantly increased survival (*P* < 0.05 and *P* < 0.001) compared to single agents in a MM xenograft model. Combined treatment also downregulated cIAP1/2 and p52 while increasing PARP cleavage in MM cells in vivo.

**Conclusions:**

Our data suggest that birinapant and bortezomib interact synergistically in MM cells, including those resistant to bortezomib, through inactivation of the non-canonical NF-κB and activation of the extrinsic apoptotic pathway both in vitro and in vivo. They also argue that a strategy combining cIAP antagonists and proteasome inhibitors warrants attention in MM.

**Electronic supplementary material:**

The online version of this article (10.1186/s13045-019-0713-x) contains supplementary material, which is available to authorized users.

## Background

Multiple myeloma (MM) is an accumulative disease of mature plasma cells that is incurable in the majority of cases [1]. Despite the development of highly effective drugs including proteasome inhibitors (e.g., bortezomib, carfilzomib), immunomodulatory agents (e.g., lenalidomide, pomalidomide), and various therapeutic CD38 antibodies (daratumumab, isatuximab, and MOR202) [2–4], therapeutic resistance and disease progression invariably supervene. One of the hallmarks of MM cells is NF-κB pathway dependence [5]. The canonical NF-κB pathway is classically activated by TNFα, whereas the non-canonical (alternative) pathway is activated by CD40 ligation and BAFF/APRIL [6]. NF-κB is frequently activated in MM [7] and represents a critical survival factor for these cells [5]. Mutations in diverse NF-κB pathway components are common in MM [8], making NF-κB a high-priority target in this disease [9]. Notably, proteasome inhibitor (e.g., bortezomib) activity has been attributed to NF-κB inhibition [9] presumably secondary to sparing of IκBα, which binds RelA and prevents nuclear translocation and activation [10].

BIRC family genes (1–8) encode inhibitor of apoptosis proteins (IAPs) including NAIP, cIAP1, cIAP2, XIAP, survivin, BRUCE/apollon, livin/ML-IAP, and IAP-like protein 2 (ILP-2), respectively. Their unifying structural motif is the baculoviral IAP repeat (BIR) domain, while other important domains (e.g., RING) have recently been identified [11]. IAPs (e.g., cIAP1/2) containing RING domains with E3 ubiquitin ligase activity trigger proteasomal degradation of proteins, including IAPs themselves [12]. Following MOMP (mitochondrial outer membrane permeabilization), mitochondrial Smac (second mitochondrial activator of caspases) undergoes cytoplasmic release where it neutralizes IAPs, promoting apoptosis [13]. IAPs are prevalent in many types of cancer, including MM [14], and increased expression is associated with chemoresistance, disease progression, and poor prognosis [14]. Moreover, pre-clinical studies suggest a role for Smac mimetics (SMs) in MM [15]. Currently, several SMs, including the monovalent IAP antagonist LCL161 [16] and the bivalent IAP antagonist birinapant (TL32711) [17] have been evaluated in humans (NCT01681368).

BCL-2 family protein deregulation in MM and the ability of IAP antagonists to activate caspases directly, thus bypassing BCL-2/MCL-1 [18], make such compound attractive candidates in this disease. Recent studies have highlighted cross talk between IAPs, and specifically cIAP1/2, in both the canonical and non-canonical NF-κB pathways, as well as the extrinsic apoptotic cascade. For example, cIAP1/2 are components of complex I containing RIP1 and TRADD implicated in canonical NF-κB pathway activation through the NEMO/IKKα/β complex [19]. Additionally, cIAP1/2 negatively regulates the extrinsic apoptotic pathway by preventing RIP1-mediated formation of pro-death complex II (the ripoptosome), consisting of RIP1, cFLIP, FADD, and caspase-8 [20]. While cIAP1/2 may directly mediate ubiquitination and degradation of NIK (NF-κB-initiating kinase), a key activator of the non-canonical NF-κB pathway, their predominant action is to promote ubiquitination/degradation of TRAF3 (TNF receptor-associated factor 3), required for NIK ubiquitination/degradation [21]. Significantly, the non-canonical NF-κB pathway plays a key role in interactions between MM cells and the microenvironment (e.g., via BAFF, APRIL, CD40), which contributes to MM drug resistance [22]. Previous studies have shown that SMs increase the activity of various conventional or targeted agents (e.g., cisplatin, TRAIL etc.) in various tumor types [15, 23]. In MM cells, cIAP2 has been implicated in bortezomib resistance [24]. Results of a recent study indicated that the Smac mimetic BV6 and bortezomib triggered cell death in B cell lymphoma cells in vitro [25]. However, mechanisms by which clinically relevant SMs interact with proteasome inhibitors are largely unknown, particularly in MM, a disease in which bortezomib represents a mainstay of therapy, nor are data available involving primary MM cells or in vivo models. Here, we report that the clinically relevant SM birinapant (TL) interacts synergistically with bortezomib (Btz) in MM cells, including Btz-resistant cells, and that this interaction involves cIAP downregulation, non-canonical pathway interruption, and extrinsic apoptotic cascade activation. Moreover, similar interactions occur in primary MM cells, including populations enriched for stem cell-like cells and in in vivo models.

## Materials and methods

### Cell lines and reagents

Human NCI-H929, U266, and RPMI8226 cells (ATCC) were maintained as before. U266 and RPMI8226 cells were authenticated (Basic STR Profiling Service, ATCC® 135-X) by ATCC immediately after this study was completed. Btz-resistant cells (PS-R) and Btz-resistant RPMI8226 (8226/V10R) sublines were maintained as described [26]. Primary bone marrow mononuclear cells were obtained with informed consent from patients with MM and analyzed as described in Additional file 1: Supplementary Materials and Methods.

All experiments used logarithmically growing cells (3–5 × 105 cells/ml). MycoAlert (Lonza, Allendale, NJ) assays were performed, demonstrating that cells were free of mycoplasma contamination.

Aminoactinomycin D (7-AAD) was purchased from Sigma/Aldrich, St. Louis, MO. Btz (Velcade®) was provided by Millennium Pharmaceuticals (Cambridge, MA, USA). TL32711 (NSC 756502; birinapant; TL) was provided by Tetralogic Pharmaceuticals (Indianapolis, IN, USA) through the Cancer Treatment and Evaluation Program (CTEP), NCI. The irreversible pan-caspase inhibitor (Z-VAD-FMK) was purchased from Selleck Chemicals (Houston, TX, USA). Drugs were dissolved in dimethyl sulfoxide (DMSO), aliquoted, and stored at − 80 °C; final DMSO concentrations were ≤ 0.1%.

### Nuclear extracts

Nuclear proteins were isolated using a Nuclear Extract kit (Active Motif, Carlsbad, CA) following the suppliers’ instructions.

### Chemiluminescent DNA-binding ELISA-based assay for activated p65 and p52

DNA binding capacity of NF-κB was determined in U266 cell nuclear extracts using the TransAM® (Active Motif) NF-κB p65 or p52 Chemi Act Assay according to the suppliers’ instructions.

### RNA interference, immunoblotting analysis, and analysis of cell death (apoptosis) and co-culture of MM cells with stromal cells

See Additional file 1: Supplementary Methods.

### Animal studies

A xenograft murine model was used. NOD/SCID-γ mice (Jackson Laboratories) were injected subcutaneously with 5 × 10^6^ U266 or bortezomib-resistant cells (PS-R) stably transfected with a construct encoding luciferase. Tumor growth was monitored weekly using calipers, and mean tumor volume was calculated using the formula (1/2 × [length × width^2^]). When mean tumor volumes reached 150–200 mm^3^ (18 days post-injection), animals were randomized into treatment groups. TL (TL32711) was dissolved in 12.5% Captisol (Ligand Pharmaceuticals) in distilled water. Bortezomib (Btz) was dissolved in DMSO and was diluted in 0.9% saline. Both were administered via intra-peritoneal (i.p.) injection of TL and Btz on days 1, 4, 8, and 11 of a cycle. Animals were each treated with two 14-day cycles. Control animals were injected with equal volumes of vehicle.

Mice were monitored for tumor growth with an IVIS 200 imaging system (Xenogen Corporation, Alameda, CA) weekly. Measurement of animal body weight was performed twice/week to monitor toxicity.

### Statistical analysis

Values represent the means ± SD for at least three independent experiments performed in triplicate. The significance of differences between experimental variables was determined using two-tailed Student’s *t* test. The significance of *P* values are *P < 0.05, **P < 0.01, ***P < 0.001, or ****P < 0.0001. Synergism was determined by the method of Chou and Talalay [27] using a commercially available software program (Calcusyn, Biosoft, Ferguson, MO). Kaplan-Meier analysis of mouse survival was performed with GraphPad Prism 6 software (La Jolla, CA), and a log-rank test (Mantel-Cox) was performed to compare survival curves.

## Results

### TL/Btz synergistically induces apoptosis in MM cells

**Fig. 1:**
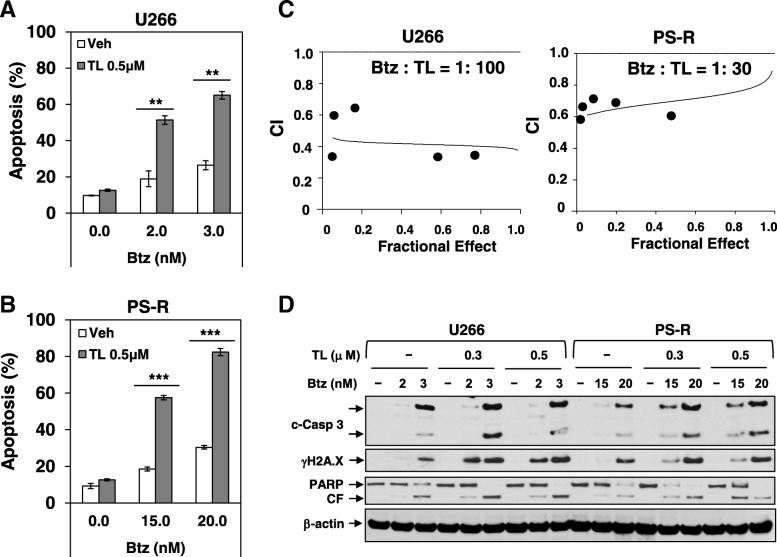
TL32711 interacts synergistically with Btz to induce apoptosis in both Btz-naïve MM cells and Btz-resistant MM cells. **a**–**b** Btz-naïve U266 cells or Btz-resistant PS-R cells were exposed to the indicated concentrations of Btz +/− TL for 24 h (U266) or 48 h (PS-R), followed by flow cytometric analysis of cell death after staining with 7-AAD. **c** U266 and PS-R cells were exposed (24 h or 48 h) to varying concentrations of Btz +/− TL at a fixed ratio (1:100 or 1:30), after which the percentage of annexin V+ cells was determined. Median dose-effect analysis was then employed to characterize the nature of the interaction between these agents with constant ratio. The fraction affected (FA) less than 1.0 reflect synergistic interactions. The results are representative of three separate experiments. **d** U266 and PS-R cells were incubated with Btz +/− TL for 24 h or 48 h, after which γH2A.X and cleavage of Caspase-3 and PARP were monitored by immunoblotting analysis. CF, cleavage fragment. β-actin was assayed to ensure equivalent loading and transfer. **P < 0.01; ***P < 0.001

Exposure (24 h) of U266 cells to low, minimally toxic concentrations of TL (0.5 μM) significantly increased the lethal effects of very low Btz concentrations (2 or 3 nM) (Fig. 1a). Similar interactions were observed in highly Btz-resistant PS-R cells, using higher Btz concentrations (e.g., 10–15 nM) and longer exposure intervals (48 h) (Fig. 1b). Median dose-effect analysis revealed combination index (CI) values less than 1.0 in each cell line, reflecting synergism (Fig. 1c). Western blot analysis of both cell lines demonstrated that combined treatment resulted in marked caspase-3/PARP cleavage and γH2A.X generation, a characteristic of DNA double-strand breaks (Fig. 1d). Time course studies revealed that changes became apparent after 16-h drug exposure (Additional file 1: Figure S1A). Synergistic interactions between TL and Btz were also observed in 8226, Btz-resistant 8226/v10R, and H929 MM cells (Additional file 1: Figure S1B–D), indicating that TL interacts synergistically with Btz in both Btz-sensitive and Btz-resistant MM cells.

### TL/Btz downregulates cIAP1/2 but does not inactivate the canonical NF-κB pathway

**Fig. 2:**
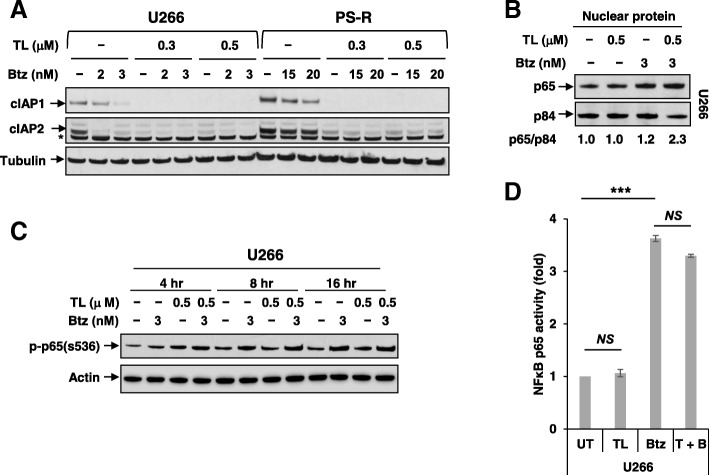
TL +/− Btz downregulates cIAP1/2 but does not inactivate the canonical NF-κB pathway. **a** U266 and PS-R cells were treated with Btz +/− TL for 24 h, after which cIAP1 and cIAP2 were monitored by immunoblotting analysis. α-Tubulin was assayed to ensure equivalent loading and transfer. **b** U266 cells were exposed to the indicated concentrations of Btz +/− TL for 16 h, after which nuclear protein was extracted from the cells. Immunoblotting analysis was then performed to monitor levels of p65. p84 was assayed to ensure equivalent loading and transfer. **c** U266 cells were incubated with 500 nM TL32711 +/− 3 nM Btz for 4 h, 8 h, and 16 h, after which p-p65 (S536) was monitored by immunoblotting analysis. β-actin was assayed to ensure equivalent loading and transfer. **d** Following treatment as in **b**, nuclear proteins were isolated using a Nuclear Extract Kit. DNA binding of NF-κB (p65 subunit) was determined using TransAM for NF-κB activity. ***P < 0.001; NS not significant

Effects of TL and Btz on cIAP expression and effects on the canonical NF-κB pathway were examined in U266 and PS-R cells. In both cell types, exposure to TL (± Btz) sharply downregulated cIAP1 expression (Fig. 2a), consistent with effects in other tumor cell types [17]. Similar results were observed in other MM cell lines (e.g., H929, OPM2) (Additional file 1: Figure S2A–B). Notably, Btz alone modestly downregulated cIAP1, in accord with previous findings [28]. Parallel reductions in cIAP2 were also observed. Interestingly, exposure (16 h) to Btz modestly increased nuclear expression of p65 in MM cells, indicating canonical pathway activation, as previously reported in lymphoma cells [29]. Significantly, this effect was not blocked by TL (Fig. 2b). Shorter exposures (e.g., 4–16 h) yielded similar results (Fig. 2c), as did the results of an NF-κB p65 Chemi Act Assay (Fig. 2d). Thus, combining TL with Btz in MM cells was associated with cIAP1/2 downregulation but not with canonical NF-κB pathway inactivation.

### The TL/Btz regimen upregulates TRAF3, downregulates TRAF2 and BCL-xL, and diminishes activation of the non-canonical pathway

**Fig. 3:**
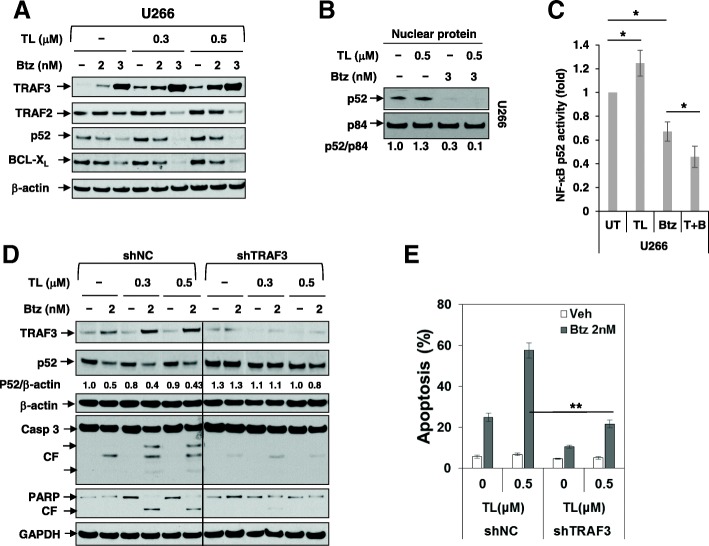
Upregulation of TRAF3 plays a functional role in TL/Btz-induced apoptosis. **a** U266 cells were treated with Btz +/− TL for 24 h, after which TRAF3, TRAF2, p52, and BCL-X_L_ were monitored by immunoblotting analysis. β-actin was assayed to ensure equivalent loading and transfer. **b** U266 cells were exposed to the indicated concentrations of Btz +/− TL for 16 h, after which nuclear protein was extracted from the cells. Immunoblotting analysis was then performed to monitor levels of p52. p84 was assayed to ensure equivalent loading and transfer. **c** DNA binding of NF-κB (p52 subunit) was determined using a TransAM assay for NF-κB activity. **d**–**e** U266 cells were stably transfected with constructs encoding shRNA targeting *TRAF3* (shTRAF3) or scrambled sequence as a negative control (shNC). Cells were treated with Btz +/− TL for 24 h, after which cell death was analyzed by flow cytometry following staining with 7-AAD (**e**). The results shown are representative of three separate experiments. Immunoblotting analysis was carried out to monitor TRAF3, p52, caspase-3, and PARP (**d**). A black line separates images acquired from different regions of the same gel with identical exposures. Densitometry analysis was performed using ImageJ. Values indicate fold-change of p52 versus untreated control (arbitrarily set as 1.0), after normalization to β-actin. CF, cleavage fragment. β-actin and GAPDH were assayed to ensure equivalent loading and transfer. *P < 0.05; **P < 0.01

Parallel studies were performed to characterize effects of the regimen on the non-canonical NF-κB pathway. Exposure (24 h) of U266 cells to Btz modestly increased expression of TRAF3, a key component of the non-canonical pathway, and TL co-administration increased this action (Fig. 3a). In contrast, co-administration of Btz and TL reduced expression of TRAF2, also a component of this pathway [30]. Similar results were observed in H929 and OPM-2 cells (Additional file 1: Figure S2A and B). Notably, IAP antagonism (e.g., by TL) modestly upregulated nuclear p52, an indicator of non-canonical pathway activation, consistent with previous reports [31]. However, co-exposure (16 h) to Btz blocked this effect (Fig. 3b). Concordant results were obtained when an NF-κB p52 activity assay was employed (Fig. 3c). These events were accompanied by a downregulation of BCL-X_L_, a downstream target of this pathway [32] (Fig. 3a), indicating that in contrast to effects on canonical NF-κB signaling, the TL/Btz regimen opposed activation of the non-canonical NF-κB pathway.

### TRAF3 upregulation contributes functionally to TL/Btz toxicity

To assess the functional significance of TRAF3 upregulation, U266 cells stably expressing TRAF3 shRNA were generated (shTRAF3). These cells exhibited clearly diminished TRAF3 induction following TL/Btz exposure accompanied by increased p52 processing compared to non-targeting controls (Fig. 3d). TL/Btz-treated shTRAF3 cells also exhibited a significant reduction in apoptosis compared to controls (**P < 0.01; Fig. 3e).

### Overexpression of TRAF2 or BCL-X_L_ diminishes TL/Btz-induced apoptosis

**Fig. 4:**
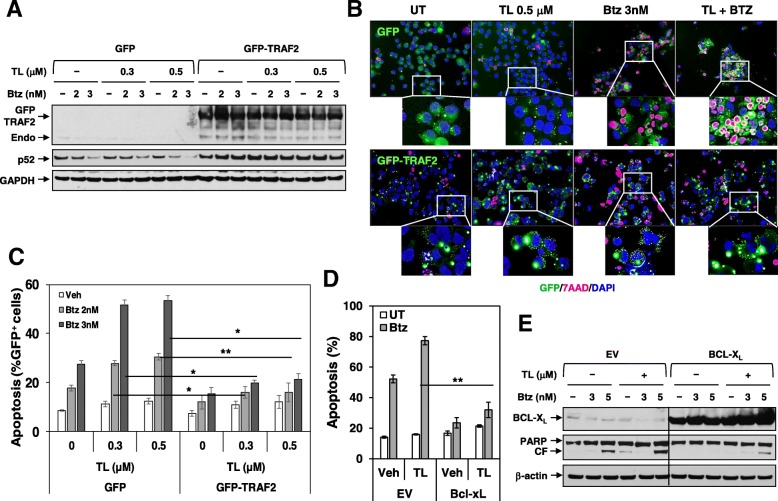
Overexpression of TRAF2 or BCL-X_L_ significantly diminishes TL/Btz-induced apoptosis. **a**–**c** U266/GFP-TRAF2 and U266/GFP cells were established by stably transfecting cells with full-length human *TRAF2* cDNA or empty vector. Cells were treated with Btz +/− TL for 24 h. **a** Immunoblotting analysis was performed to monitor TRAF2 and p52. GAPDH was assayed to ensure equivalent loading and transfer. Endo, endogenous. **b** Cytospin slides were prepared, stained with 7-AAD, and counterstained with DAPI. Images were obtained with an IX71-Olympus inverted system microscope at × 200 magnification. **c** After drug treatment, cells were subjected to flow cytometry to determine the percentage of dead (7-AAD^+^) cells in GFP^+^ cells (*P < 0.05; **P < 0.01). Values represent the means ± SD for at least three independent experiments performed in triplicate. **d**–**e** U266/BCL-X_L_ and U266/EV cells were established by stably transfecting full-length human BCL-X_L_ cDNA or empty vector. Cells were treated with Btz +/− TL for 24 h. After drug treatment, cells were subjected to flow cytometry to determine the percentage of dead (7-AAD^+^) cells (**P < 0.01). Values represent the means ± SD for at least three independent experiments performed in triplicate. **e** Immunoblotting analysis was performed to monitor BCL-X_L_ and PARP. A black line separates images acquired from different regions of the same gel with identical exposures. CF, cleavage fragment. β-actin was assayed to ensure equivalent loading and transfer

To evaluate the functional significance of TRAF2 and BCL-X_L_ downregulation, U266 cells ectopically expressing TRAF2 (GFP-TRAF2) were generated (Fig. 4a). Following TL/Btz treatment, GFP-TRAF2 cells exhibited markedly diminished p52 downregulation compared to controls. Fluorescence microscopy revealed sharply reduced apoptosis (7-AAD, red staining) in TRAF2 cells compared to controls following combined drug exposure (Fig. 4b). Quantification documented highly significant reductions in cell death (Fig. 4c). Cells ectopically expressing BCL-X_L_ were also very significantly protected from TL/Btz-induced apoptosis (**P < 0.01; Fig. 4d) and PARP cleavage (Fig. 4e) compared to controls, indicating that TRAF2/BCL-X_L_ downregulation and TRAF3 upregulation contribute functionally to TL/Btz toxicity in MM cells.

### Blockade of FADD diminishes TL/Btz-induced apoptosis

**Fig. 5:**
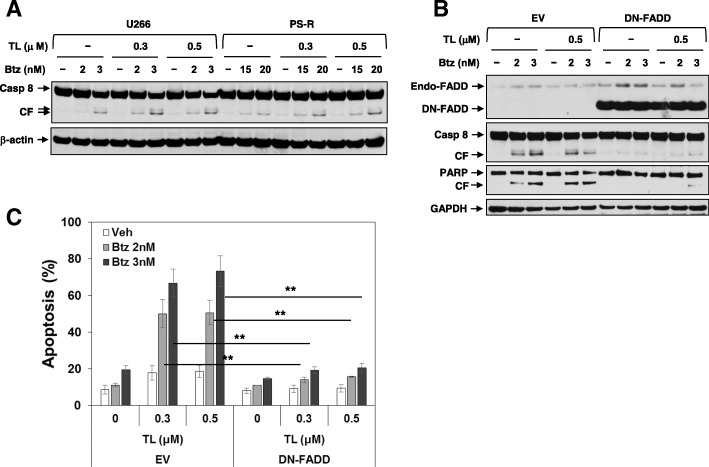
Blockade of FADD significantly reduces TL/Btz-induced apoptosis. **a** U266 and PS-R cells were treated with Btz +/− TL for 24 h. Immunoblotting analysis was carried out to monitor caspase-8 expression. CF cleavage fragment. β-actin was assayed to ensure equivalent loading and transfer. **b**–**c** U266/DN-FADD and U266/EV cells were established by stably transfecting human dominant-negative FADD cDNA or empty vector. Cells were treated with Btz +/− TL for 24 h. **c** After drug treatment, cells were subjected to flow cytometry to determine the percentage of dead (7-AAD^+^) cells (**P < 0.01). Values represent the means ± SD for at least three independent experiments performed in triplicate. Immunoblotting analysis was performed to monitor FADD, caspase-8, and PARP expression. Endo, endogenous; CF. cleavage fragment. GAPDH was assayed to ensure equivalent loading and transfer

The death-inducing signaling complex (DISC), comprised of Fas, FADD, and caspase-8, represents a component of the extrinsic apoptotic pathway [33]. Given evidence that cIAPs negatively regulate the extrinsic apoptotic pathway [34], the functional role of extrinsic pathway activation on TL/Btz anti-MM activity was examined. Both U266 and Btz-resistant PS-R cells displayed sharply increased caspase-8 cleavage following TL/Btz exposure (Fig. 5a). To determine the functional role of this phenomenon, U266 cells ectopically expressing dominant-negative FADD were employed (DN-FADD). These cells displayed dramatically reduced caspase 8 and PARP cleavage compared to controls (Fig. 5b). Consistently, DN-FADD expression significantly protected U266 cells from TL/Btz-induced apoptosis (**P < 0.01; Fig. 5c). Identical results were obtained in cells expressing dominant-negative caspase 8 (Additional file 1: Figure S3A–B). Furthermore, the pan-caspase-inhibitor Z-VAD-FMK essentially abrogated caspase-3 cleavage induced by TL/BTZ but not cIAP1/2 downregulation (Additional file 1: Figure S3C). Together, these findings argue that extrinsic pathway activation contributes functionally to TL/Btz activity.

### TL/Btz circumvents microenvironment-driven intrinsic resistance

**Fig. 6:**
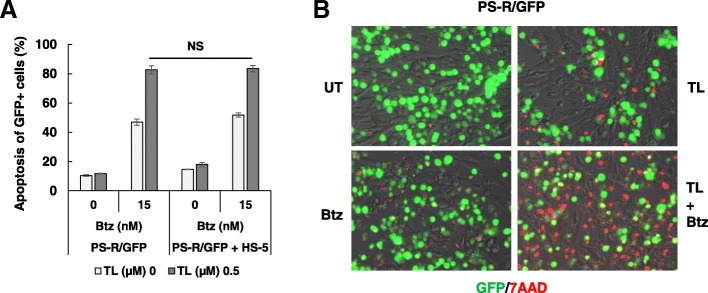
TL/Btz circumvents microenvironment-driven intrinsic resistance. **a** GFP-labeled PS-R cells co-cultured with or without BM stromal HS-5 cells and were incubated with Btz +/− TL for 48 h. Apoptosis of GFP^+^ cells was analyzed by multi-color flow cytometry of 7-AAD staining. **b** Cells were stained with 7-AAD to monitor death of GFP^+^ cells. Bright field images were captured with an IX71-Olympus inverted system microscope at × 200 magnification. NS, not significant

HS-5 co-culture studies were performed to determine whether stromal factors ameliorated TL/BTZ toxicity. Co-culture of GFP-labeled PS-R cells with HS-5 cells failed to protect cells following 40 h TL/Btz exposure (Fig. 6a). Fluorescence microscopy revealed a marked increase in red staining (7-AAD uptake) after drug treatment in GFP-labeled PS-R cells cultured in the presence of HS-5 cells (Fig. 6b). To further determine whether growth factors (e.g., IL-6 or vascular endothelial growth factor (VEGF)) ameliorated TL/Btz toxicity, cell viability and cell death assays were performed. Co-culture of U266 cells with IL-6 or VEGF failed to protect cells from treatment of TL/Btz (Additional file 1: Figure S4).

### TL/Btz is active against primary CD138^+^ MM cells and diminishes the primitive progenitor cell-enriched CD138^−^/CD19^+^/CD20^+^/CD27^+^ population while sparing normal CD34^+^ cells

**Fig. 7:**
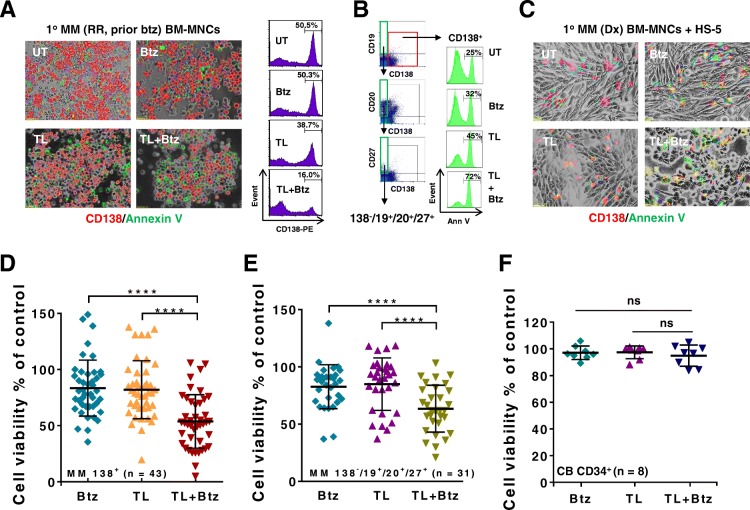
The TL/Btz regimen is active against primary CD138^+^ MM cells and diminishes primitive progenitor cell-enriched CD138^−^/CD19^+^/CD20^+^/CD27^+^ populations while sparing normal CD34^+^ cells. **a** Representative primary bone marrow cells from a patient with MM (RR, relapse and refractory; prior Btz) were exposed to 500 nM TL +/− 3 nM Btz for 24 h, after which the cells were stained with CD138-PE and annexin V-FITC. Images were obtained with an IX71-Olympus inverted system microscope at × 200 magnification. Flow cytometric analysis was performed to determine the CD138^+^ population. **b** After exposure to 500 nM TL +/− 3 nM Btz for 24 h, the percentage of primitive CD138^−^/CD19^+^/CD20^+^/CD27^+^ cells in bone marrow mononuclear cells from a primary MM sample was determined by multi-color FCM. **c** Primary cells from a patient with newly diagnosed MM were exposed to 500 nM TL +/−  3 nM Btz for 24 h in the presence of HS-5, after which they were stained with CD138-PE and annexin V-FITC. Images were captured with an IX71-Olympus inverted system microscope at × 200 magnification. **d** Parallel experiments were carried out with 43 primary samples. Viability of CD138^+^ cells was analyzed by multi-color flow cytometry determination of 7-AAD. Lines indicate means and SD (****P < 0.001). **e** After exposure to 500 nM TL +/− 3 nM Btz for 24 h, apoptosis of CD138^−^/CD19^+^/CD20^+^/CD27^+^ cells (*n* = 31) was analyzed by 7-AAD staining and quantitated by multi-color flow cytometry (****P < 0.001). **f** Parallel experiments were carried out with eight primary cord blood (CB) CD34^+^ samples. ns, not significant

Parallel studies were performed in primary CD138^+^ MM cells. Bone marrow (BM) mononuclear cells obtained from a patient with relapsed/refractory MM who had progressed while receiving Btz were incubated (24 h) with 2 nM Btz ± 0.5 μM TL, after which they were stained with antibodies directed against CD138 (red) and annexin V (green). As shown by the light microscopic images in Fig. 7a (left panels), Btz alone exerted little effect while TL modestly increased the number of annexin V^+^ cells. However, combined treatment sharply increased green staining and reduced the number of CD138^+^ cells. Flow cytometric analysis (Fig. 7a, right panels) confirmed these findings. Parallel results were obtained when a more primitive sub-population of MM cells (CD138^−^/CD19^+^/CD20^+^/CD27^+^) [35] was analyzed (Fig. 7b). In addition, primary cells from a newly diagnosed MM patient were exposed to TL and Btz in the presence of HS-5 cells, followed by staining for CD138^+^ expression and annexin V positivity as above. Co-exposure of cells to TL and Btz induced a pronounced increase in green annexin V^+^ cells and the virtual loss of CD138^+^ cells despite the presence of HS-5 (Fig. 7c).

Analogous studies were performed on 43 primary MM samples (Additional file 2: Table S1). Whereas exposure to TL (0.5 μM, 24 h) or Btz (2 nM, 24 h) individually modestly diminished the mean survival of these specimens, combined treatment very significantly reduced survival compared to single-agent exposure (*P* < 0.0001 in each case; Fig. 7d). Comparable results were obtained in the sub-set of samples (*n* = 31) in which the CD138^−^/CD19^+^/CD20^+^/CD27^+^ population could be detected (****P < 0.0001; Fig. 7e). Similar results were seen in patient sub-group analysis involving newly diagnosed and relapsed/refractory MM patients (Additional file 1: Figure S5A–B, *P < 0.05, ***P < 0.001, ****P < 0.0001, respectively). Reductions in primary CD138-PE-staining cells are illustrated in the photomicrographs shown in Additional file 1: Figure S6A–B involving drug-naïve and Btz-refractory primary samples. In contrast to these findings, identical exposure to TL and Btz was non-toxic to eight normal cord blood CD34^+^ cells (*P* > 0.05 versus untreated controls; Fig. 7f). These findings argue that the TL/Btz regimen targets primary MM cells, including BTZ-resistant cells, as well as more primitive MM sub-populations, while exhibiting minimal toxicity toward normal hematopoietic progenitors. They also indicate that this regimen circumvents the cytoprotective effects of microenvironmental factors.

### TL/Btz suppresses tumor growth in a murine xenograft model

**Fig. 8:**
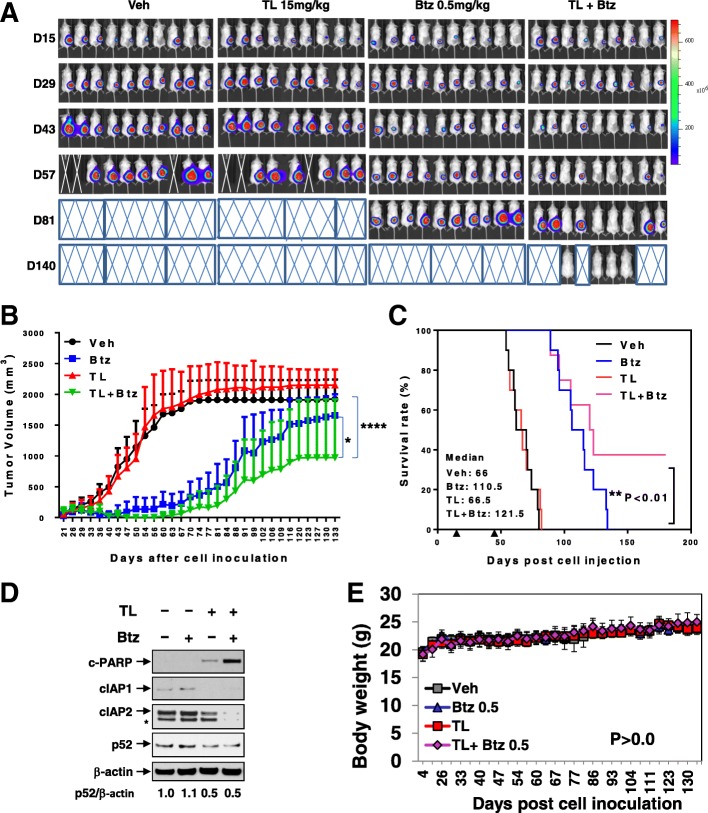
Co-administration of TL and Btz suppresses tumor growth in a MM xenograft model. **a**–**d** NOD/SCID-γ (NSG) mice were subcutaneously (s.c.) inoculated in the right rear flank with 5 × 10^6^ luciferase-expressing U266 cells. TL and Btz were administered via intra-peritoneal (i.p.) injection at a dose of 15 mg/kg (TL) and 0.5 mg/kg (Btz). **a** Tumors were monitored every other day after i.p. injection with 150 mg/kg luciferin using an IVIS 200 imaging system. Mice were euthanized when tumor length reached 17 mm or humane endpoints were reached; Veh, vehicle. **b** Tumor size was measured every other day. *P < 0.05 vs Btz; ****P < 0.0001 vs TL. **c** Kaplan-Meier analysis was carried out to analyze survival. Inset, median survival days. Arrows indicate the time when treatment began (day 18) and was discontinued (day 48). **P < 0.01 vs Btz. **d** Western blot analysis was performed to monitor the indicated candidate proteins, identified from in vitro studies, in tumors excised from representative mice. Densitometry analysis was performed using ImageJ. Values indicate fold-change of p52 versus untreated control (arbitrarily set as 1.0), after normalization to β-actin. **e** Mice did not display significant body weight loss (≥ 20%) compared to initial weight (*P* > 0.05 vs each single agent) or other signs of toxicity over the course of treatment

Finally, the in vivo effects of the regimen were evaluated in a mouse flank MM xenograft model. Following inoculation with luciferase-labeled U266 cells, animals were treated with TL (15 mg/kg) ± Btz (0.5 mg/kg) by i.p. injection, after which tumor volumes were measured two times per week and tumor growth monitored by IVIS imaging. Combined treatment clearly reduced tumor burden compared to single-agent treatment (Fig. 8a). Tumor volumes were also significantly diminished compared to individual treatment by combined TL/Btz exposure (****P < 0.0001 vs TL, *P < 0.05 vs Btz; Fig. 8b). Co-administration also significantly increased animal survival compared to single drug treatment (**P < 0.01 vs Btz, log-rank (Mantel-Cox) test; Fig. 8c). Western blot analysis performed on tumor specimens revealed that combined treatment triggered enhanced PARP cleavage, cIAP downregulation, and diminished p52 processing (Fig. 8d). Parallel studies were performed utilizing bortezomib-resistant PS-R cells and showed similar reductions in tumor growth with the combination (Additional file 1: Figure S7). Finally, TL/Btz treatment induced minimal toxicity and weight loss (*P* > 0.05, Fig. 8e; Additional file 1: Figure S7E), indicating that the TL/Btz regimen is effective and tolerable in animals, and raising the possibility that mechanisms observed in vitro may be operative in vivo.

## Discussion

By bypassing inhibition of activated caspases by IAP family members (e.g., XIAP) [36], SMs increase the activity of both conventional and novel cytotoxic agents [37], presumably by lowering the apoptotic threshold. Recent attention has focused on the role of cIAP1/2 in governing the activity of pathways relevant to MM cell survival, including the non-canonical NF-κB pathway [8] and the extrinsic apoptotic cascade [38]. In this context, cIAP1 plays a complex role in regulating the activity of the non-canonical NF-κB pathway [21], which has been implicated in microenvironmental forms of resistance in malignant B cells [24, 39]. In addition, cIAP1 inhibits the ripoptosome (complex II), protecting cells from the lethal consequences of extrinsic apoptotic pathway activation [20]. Such findings argue that perturbations in these pathways contribute functionally to IAPi/PI anti-myeloma activity and that this strategy may be effective in the setting of Btz or stromal factor-related forms of resistance.

The marked cIAP1 downregulation in TL/Btz-treated cells is counterintuitive given the mechanisms of the action of these agents. For example, the E3 ubiquitin ligase activity of the RING domain of cIAP1 triggers ubiquitination and subsequent proteasomal degradation of multiple proteins, including IAPs themselves [12]. Nevertheless, the ability of SMs to downregulate cIAP1 is well described and has been attributed to E3 ligase activation requiring binding to TRAF2 and E2 [40]. In addition, PIs might be expected to antagonize the proteasomal elimination of cIAP. However, in all MM cell types examined, combined TL/Btz exposure was associated with pronounced cIAP downregulation, arguing, albeit indirectly, that non-proteasomal mechanisms are responsible for cIAP downregulation in this setting.

Contrary to expectations, the TL/Btz regimen did not inactivate the canonical NF-κB pathway. While proteasome inhibitors such as Btz have classically been thought to inactivate NF-κB by preventing elimination of the NF-κB-inhibitory molecule IκBα [41, 42], this has not been a universal phenomenon. For example, Btz has been shown to activate, rather than inhibit NF-κB signaling in MM and lymphoma cells [29], possibly by triggering autophagic IκBα degradation. The results of the present studies revealed that combining Btz with TL increased phosphorylation and nuclear accumulation of p65, arguing that interruption of the canonical NF-κB does not underlie the anti-myeloma activity of the TL/Btz regimen.

In contrast, the TL/Btz regimen diminished non-canonical pathway activation, and this phenomenon contributed significantly to the regimen’s toxicity. In this regard, IAP antagonists have been referred to as “double-edged swords”/tumor suppressors [43], as cIAP1 is involved in degradation of downstream non-canonical pathway components [21]. However, cIAP inhibitors also upregulate the TRAF3-dependent E3 ubiquitin ligase, which opposes such effects [44]. Notably, combined TL/Btz exposure induced marked TRAF3 accumulation, and TRAF3 knockdown significantly reduced TL/Btz toxicity. Such findings argue that the pro-apoptotic effects of TRAF3 upregulation by IAP antagonists predominate in this setting. Additionally, combined TL/Btz exposure downregulated TRAF2 and BCL-X_L_, and both implicated in non-canonical NF-κB signaling [30, 32]. The observation that both TRAF2 and BCL-X_L_ overexpression significantly protected cells from the regimen argues that their downregulation contributed to the regimen’s activity.

Inactivation of the non-canonical pathway by TL/Btz was also associated with circumvention of microenvironmental factor pro-survival effects. Multiple studies have highlighted links between BAFF/APRIL-related activation of the non-canonical pathway and microenvironmental forms of drug resistance, particularly in malignant B cells [45]. The importance of stromal cells in conferring drug resistance in MM is also well established [46]. In accord with these findings, co-culture of MM cells with soluble factors (IL-6 or VEGF) or stromal cells failed to diminish TL/Btz toxicity, consistent with the regimen’s ability to disrupt alternative NF-κB pathway signaling.

Recent attention has focused on cross talk between components of the NF-κB pathway, particularly cIAP1/2, and activation of the extrinsic apoptotic cascade [47]. For example, cIAP1/2 negatively regulates the extrinsic apoptotic pathway by preventing RIP1-mediated formation of pro-death complex II (the ripoptosome), consisting of RIP1, cFLIP, FADD, and caspase-8 [20]. Consistent with these observations, the TL/Btz regimen robustly triggered caspase-8 activation. Significantly, regimen toxicity was markedly reduced in cells expressing dominant-negative FADD or caspase-8, indicating that extrinsic cascade activation played an important functional role in cell death. Such findings are consistent with previous reports indicating that concomitant activation of the extrinsic pathway dramatically amplifies the lethal effects of intrinsic pathway induction [48] and that this phenomenon may be particularly relevant to MM cells [47].

Importantly, the TL/Btz regimen was active against primary CD138^+^ MM cells, but identical exposures were minimally toxic to normal hematopoietic progenitors. The basis for selectivity of this regimen remains to be determined, but may reflect preferential killing of neoplastic versus normal cells by proteasome inhibitors [49], the increased dependence of the former on an intact NF-κB pathway for survival [5], and selective targeting of neoplastic cells by IAP antagonists [31]. Interestingly, more primitive MM cell progenitors (e.g., CD138^−^,CD19^+^, CD20^+^,CD27^+^) [35] were at least, if not more, sensitive to this regimen than their more mature counterparts. This stands in contrast to standard cytotoxic agents, to which primitive, quiescent cells are generally more resistant [35]. Notably, as with continuously cultured lines, stromal cells failed to protect primary CD138^+^ MM cells from the TL/Btz regimen and were also fully active against Btz-resistant cells expressing upregulation of MCL-1 and downregulation of BIM [26]. The latter capacity may reflect, at least in part, the ability of IAP antagonists to bypass apoptosis inhibition conferred by perturbations in BCL-2 family members through direct activation of caspases [36].

The present findings demonstrate that TL and Btz co-administration was well tolerated in a murine xenograft MM model, and resulted in significantly greater tumor growth inhibition and animal survival than the agents alone in both Btz-naïve MM cells and Btz-resistant MM cells. The observation that tumor cells obtained from mice displayed several of the in vitro findings (e.g., inactivation of the non-canonical NF-κB pathway, downregulation of cIAP1/2) suggests that analogous mechanisms are operative in vivo. These results differ from those of a recent study in which combining Btz with another IAP antagonist (LCL-161) in a murine MM model did not result in survival benefit [50]. This discrepancy may reflect multiple factors, including IAP antagonist-specific actions, the different model systems employed, or potentially immunologic actions of SMs [51]. In our study, we speculate that birinapant activates the non-canonical NF-κB pathway as a compensatory cytoprotective action. However, bortezomib may block birinapant-activated non-canonical NF-κB signaling, thereby diminishing this protective effect and further enhancing cell death. Nevertheless, the present findings argue that a mechanism-based strategy involving concomitant cIAP1 and proteasome antagonism warrants attention in MM, particularly in the setting of Btz refractoriness or other forms of drug resistance (e.g., microenvironmental). Currently, given recent trials in MM combining IAP antagonists (e.g., LCL161) with cytotoxic agents (e.g., Cytoxan; NCT01955434), the concept of combining birinapant with proteasome inhibitors such as bortezomib warrants consideration. Efforts to develop this strategy further are currently underway.

## Conclusions

Our data suggest that birinapant and bortezomib interact synergistically in MM cells, including those resistant to bortezomib, through inactivation of the non-canonical NF-κB and activation of the extrinsic apoptotic pathway both in vitro and in vivo. They also argue that a strategy combining cIAP antagonists and proteasome inhibitors warrants attention in MM.

## Additional files

Additional file 1: Supplementary Materials and Methods. **Figure S1.** TL +/− Btz co-administration induces apoptosis in Btz-naïve or Btz-resistant multiple myeloma cells. **Figure S2.** Btz +/− TL co-administration upregulates TRAF3 and downregulates cIAP1/2 and TRAF2 in additional multiple myeloma cell lines. **Figure S3.** Inhibition of caspase-8 significantly diminishes TL/Btz-induced apoptosis. **Figure S4.** The TL/Btz regimen is active against MM cells in the presence of IL6 or VEGF. **Figure S5.** The TL/Btz regimen is active against primary CD138^+^ MM cells and diminishes primitive progenitor cell-enriched CD138^−^/CD19^+^/CD20^+^/CD27^+^ populations while sparing normal CD34^+^ cells in both newly diagnosed and relapsed/refractory patients. **Figure S6.** The TL/Btz regimen is active against primary CD138^+^ MM cells obtained from Btz-naïve or resistant patients. **Figure S7.** Co-administration TL and Btz suppresses tumor growth in a Btz-resistant MM xenograft model. (DOCX 8528 kb)

Additional file 2: **Table S1.** Analogous studies were performed on 43 primary MM samples. (XLSX 11 kb)

## Abbreviations

7-AAD: 7-Aminoactinomycin D
BCL-X_L_: Bcl-2-like protein 1
BM: Bone marrow
Btz: Bortezomib
CIAP1/2: Cellular inhibitor of apoptosis protein-1/2
FADD: Fas-associated via death domain
IAP: Inhibitor of apoptosis protein
ILP-2: IAP-like protein 2
MCL-1: Myeloid cell leukemia sequence 1
ML-IAP: Melanoma inhibitor of apoptosis protein
MM: Multiple myeloma
NAIP: NLR family apoptosis inhibitory protein
NF-κB: Nuclear factor-kappa B
NIK: NF-kB-inducing kinase
TNF: Receptor-associated factor
TRAF: TNF receptor-associated factor
SMAC: Second mitochondrial activator of cell death
VEGF: Vascular endothelial growth factor
XIAP: X-linked inhibitor of apoptosis protein
