# Supplementary material for: The IAP antagonist birinapant potentiates bortezomib anti-myeloma activity in vitro and in vivo
Source: J Hematol Oncol. 2019 Mar 7;12:25. doi: 10.1186/s13045-019-0713-x (PMC6407248; doi:10.1186/s13045-019-0713-x)
Supplement: Supplementary file 1 — Supplementary Materials and Methods. Figure S1. TL +/− Btz co-administration induces apoptosis in Btz-naïve or Btz-resistant multiple myeloma cells. Figure S2. Btz +/− TL co-administration upregulates TRAF3 and downregulates cIAP1/2 and TRAF2 in additional multiple myeloma cell lines. Figure S3. Inhibition of caspase-8 significantly diminishes TL/Btz-induced apoptosis. Figure S4. The TL/Btz regimen is active against MM cells in the presence of IL6 or VEGF. Figure S5. The TL/Btz regimen is active against primary CD138+ MM cells and diminishes primitive progenitor cell-enriched CD138−/CD19+/CD20+/CD27+ populations while sparing normal CD34+ cells in both newly diagnosed and relapsed/refractory patients. Figure S6. The TL/Btz regimen is active against primary CD138+ MM cells obtained from Btz-naïve or resistant patients. Figure S7. Co-administration TL and Btz suppresses tumor growth in a Btz-resistant MM xenograft model. (DOCX 8528 kb) [file 13045_2019_713_MOESM1_ESM.docx]

**Supplementary Materials and Methods**

**RNA interference**

SureSilencing shRNA plasmids (neomycin resistance) targeting human TRAF3 (shTRAF3), or scrambled sequence as negative control (shNC) were purchased from Qiagen, Germantown, MD. Cells were stably transfected using the Amaxa Nucleofector (Amaxa GmbH, Cologne, Germany) device with Cell Line Specific Nucleofector Kit C (Amaxa GmbH, Cologne, Germany) as per the manufacturer's instructions. Clones with down-regulated TRAF3 or shNC were selected with 400 μg/ml G418.

**Immunoblot analysis**

Samples were prepared from whole-cell pellets. Total protein or nuclear protein were quantified using Coomassie Protein Assay Reagent (Pierce ThermoFisher Scientific, Rockford, IL). Equal amounts of protein (20µg) were separated by SDS-PAGE and electro-transferred onto nitrocellulose membrane. Where indicated, the blots were probed or re-probed with antibodies against β-actin (Sigma), GAPDH (Abcam) or α-tubulin (Oncogene Inc., San Diego, CA), to ensure equal loading and transfer of proteins. The following primary antibodies were used: Caspase-3, FADD (BD Biosciences, San Jose, CA); cleaved Caspase-3 (Asp175), cleaved PARP (Asp214), P100/P52, P65, phospho-P65 (S536), BCL-X_L_ (Cell Signaling, Beverly, MA); TRAF2, TRAF3 (Santa Cruz, Dallas, TX); CIAP1/2 (R&D, Minneapolis, MN); Caspase-8 (Enzo Life Sciences, Farmingdale, NY).

**Immunofluorescence**

Cytospin slides were fixed in 4% paraformaldehyde for 1 hr, permeabilized in 0.25% Triton X-100 in PBS, and blocked with PBS containing 1% BSA and 2% FBS. For immunofluorescent staining of cleaved-Caspase-3 (Cell Signaling), secondary Alexa Fluor 488 or Alexa Fluor 594-conjugated antibodies (Cell Signaling) were employed. Slides were mounted using DAPI (4’,6-diamidino-2-phenylindole.) Fluoromount-G (Southern Biotech, Birmingham, AL). Images were captured using an Olympus IX71 Inverted System Microscope with a DP73; 17MP color camera.

**Co-culture of MM cells with stromal cells**

The human stromal cell line HS-5 was obtained from ATCC. HS-5 cells were maintained in RPMI1640 medium containing 10% FBS and sub-cultured twice weekly by trypsinization at a subcultivation ratio of 1:5-1:6. For co-culture experiments, HS-5 cells were cultured for 6-8 h prior to seeding MM cells.

**Analysis of cell death**

Apoptosis was evaluated by flow cytometry utilizing Annexin V-FITC/PI or 7-AAD staining as previously reported [1].

For CD138-/CD19+/CD20+/CD27+ analysis, CD138- mononuclear cells isolated from primary MM bone marrows were blocked by TruStain FcX on ice for 10min, stained with C19-PE/Cy7, CD20-APC/Cy7 and/or CD27-APC (Biolegend, San Diego, CA) on ice for 30min followed by staining with Annexin V- FITC at room temperature for 15min. The percentage of apoptotic (Annexin V+) cells in the CD138-/CD19+/CD20+/27+ population was then determined using a FACSCanto flow cytometer (BD Biosciences).

**Cell viability assay**

Cell proliferation was determined by CellTiter ‐Glo luminescence cell viability assay (G7570; Promega, Madison, WI, US) in accordance to the manufacturer’s instructions.

**Supplementary Figure legends**

**Figure S1** TL +/- Btz co-administration induces apoptosis in Btz-naïve or Btz-resistant multiple myeloma cells. (A) Human myeloma U266 cells were incubated with 500 nM TL +/- 3 nM bortezomib for 4 hr, 8 hr, and 16 hr, after which cleavage of Caspase-3 and PARP was monitored by immunoblotting analysis. CF = cleavage fragment. GAPDH was assayed to ensure equivalent loading and transfer. (B-D) Human myeloma Btz-naïve 8226 cells and H929 cells (B, D), and Btz-resistant 8226/v10R cells were exposed (24 hr) to varying concentrations of Btz +/- TL at a fixed ratio (8226, 1.2:100; H929, 1:100; 8226/v10R, 1:25), after which the percentage of Annexin V+ cells were determined. Median Dose-Effect analysis was then employed to characterize the nature of the interaction between these agents. Combination Index (C.I.) values less than 1.0 denote a synergistic interaction. The results are representative of three separate experiments.

**Figure S2** Btz +/- TL co-administration up-regulates TRAF3, and down-regulates cIAP1/2 and TRAF2 in additional multiple myeloma cell lines. (A-B) H929 cells and OPM2 were treated with Btz +/- TL for 24 hr, after which TRAF3, TRAF2, cIAP1 and cIAP2 were monitored by immunoblotting analysis. β-actin and GAPDH were assayed to ensure equivalent loading and transfer.

**Figure S3**. Inhibition of Caspase-8 significantly diminishes TL/Btz-induced apoptosis. U266/DN-caspase-8 and U266/EV cells were established by stably transfecting human dominant negative caspase cDNA or empty vector. Cells were treated with Btz +/- TL for 24 hr. (A) Immunoblotting analysis was performed to monitor cleavage of Caspase-3, Caspase 8 and PARP. CF = cleavage fragment. GAPDH was assayed to ensure equivalent loading and transfer. (B) After drug treatment, cells were subjected to flow cytometry to determine the percentage of dead (7-AAD^+^) cells. Values represent the means ± SD for at least three independent experiments performed in triplicate. *P<0.05; **P<0.01. (C) U266 cells were treated for 16 h with Btz +/- TL in the presence or absence 5 µM Z-VAD-FMK. Immunoblotting analysis was performed to monitor cIAP1/2, cleavages of caspase-8 and caspase-3. β-actin was assayed to ensure equivalent loading and transfer.

**Figure S4** The TL/Btz regimen is active against MM cells in the presence of IL6 or VEGF. U266 cells co-cultured with or without IL-6 (50 ng/ml) or VEGF (50 ng/ml), and were incubated with Btz +/- TL for 48 hr. (A) Cell viability was determined by CellTiter ‐Glo luminescence cell viability assay in accordance to the manufacturer’s instructions. (B) Cells were subjected to flow cytometry to determine the percentage of dead (7-AAD^+^) cells. Values represent the means ± SD for at least three independent experiments performed in triplicate. ns: not significant.

**Figure S5** The TL/Btz regimen is active against primary CD138^+^ MM cells and diminishes primitive progenitor cell-enriched CD138^-^/CD19^+^/CD20^+^/CD27^+^ populations while sparing normal CD34^+^ cells in both newly diagnosed and relapsed/refractory patients. Patient subgroup analysis of newly diagnosed and relapsed/refractory patients was performed.

**Figure S6** The TL/Btz regimen is active against primary CD138^+^ MM cells obtained from Btz-naïve or resistant patients. (A-B) Isolated bone marrow mononuclear cells from a newly diagnosed patient (DX; A) and a relapsed/refractory patient (RR; B) were exposed to indicated concentrations of Btz +/- TL for 24 hr, after which cells were stained with CD138-PE (upper panels in each Figure; lower panels present merged with bright field). Images were obtained with an IX71-Olympus inverted system microscope at 200x magnification.

**Figure S7** Co-administration TL and Btz suppresses tumor growth in a Btz- resistant MM xenograft model. NOD/SCID-γ (NSG) mice were subcutaneously (s.c.) inoculated in the right rear flank with 5×10^6^ luciferase-expressing PS-R cells. TL and Btz were administered via intra-peritoneal (i.p.) injection at a dose of 15mg/kg (TL) and 1.0 mg/kg (Btz). (A) Tumors were monitored every other day after i.p. injection with 150 mg/kg luciferin using an IVIS 200 imaging system. Mice were euthanized when tumor length reached 17 mm or humane endpoints were reached; Veh, vehicle. (B) Tumor signal was quantified by mean luciferase activity (photons/sec/cm^2^/sr) at day 30. ***P<0.001 TL+PS vs Btz; ***P<0.001 TL+PS vs TL. (C) Tumor size was measured every other day. Tumor volume was shown at day 30. *P<0.05 TL+PS vs Btz; *P<0.05 TL+PS vs TL. (D) Tumors were removed and weighed at day 33 after cell injection. P=0.032 TL+PS vs Btz; P=0.036 TL+PS vs TL. (E) Mice did not display significant body weight loss (≥20% of initial weight) or other signs of toxicity due to treatment. (F) Western blot analysis was performed to monitor the indicated candidate proteins, identified from *in vitro* studies, in tumors excised from representative mice. Densitometry analysis was performed using ImageJ. Values indicating fold-change of p52 versus untreated control (arbitrarily set as 1.0), after normalization to β-actin.

1. Dai Y and Grant S. Methods to study cancer therapeutic drugs that target cell cycle checkpoints. Methods Mol Biol. 2011;782:257-304.
